# Supplementary figures and images for: Posttranslational modification of the RHO of plants protein RACB by phosphorylation and cross-kingdom conserved ubiquitination
Source: PLoS One. 2022 Mar 25;17(3):e0258924. doi: 10.1371/journal.pone.0258924 (PMC8956194; doi:10.1371/journal.pone.0258924)

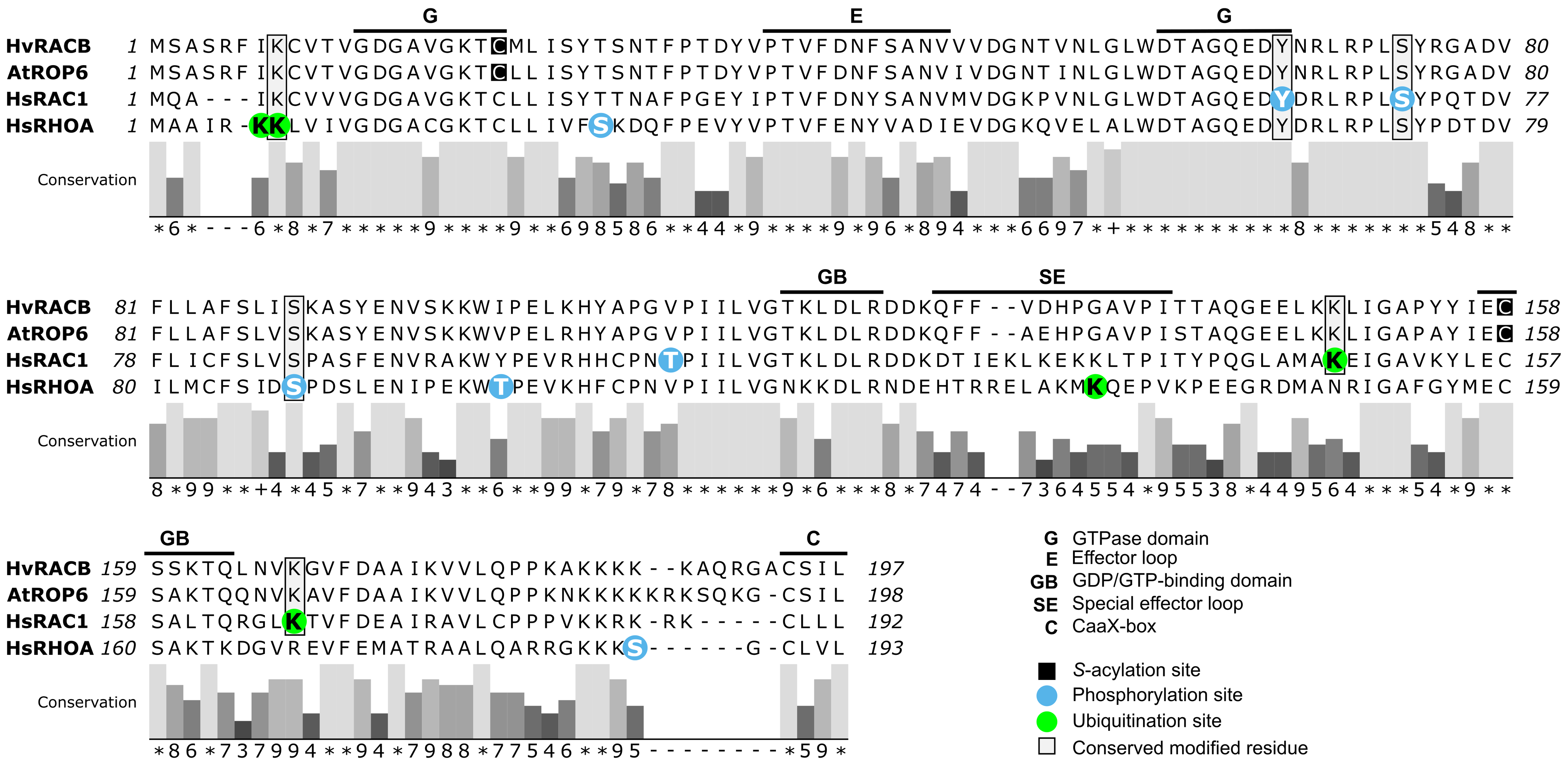

Supplement: S1 Fig — Previously published phosphorylation (blue) and ubiquitination (green) sites of mammalian Rac1 and RhoA are highlighted here in the human RAC1 and RHOA sequences. Conserved sites are highlighted in grey boxes. Additionally, the GTPase domain (G), effector loop (E), nucleotide-binding domain (GB), special effector loop (SE) and CaaX-box prenylation motif (C) are illustrated and were taken from [28]. AtROP6 has been shown to be transiently S-acylated at two cysteines [64, 65], which are also conserved in RACB (black boxes). Conservation of amino acids was determined using MAFFT multiple-sequence alignment in Jalview [70, 71]. Conservation scores range from 0 to 11 and are shown as bars (dark grey = less conserved, light grey = highly conserved). Scores of 0, 10, and 11 are shown as “-“, “+” and “*”, respectively). (TIF) [file pone.0258924.s001.tif]

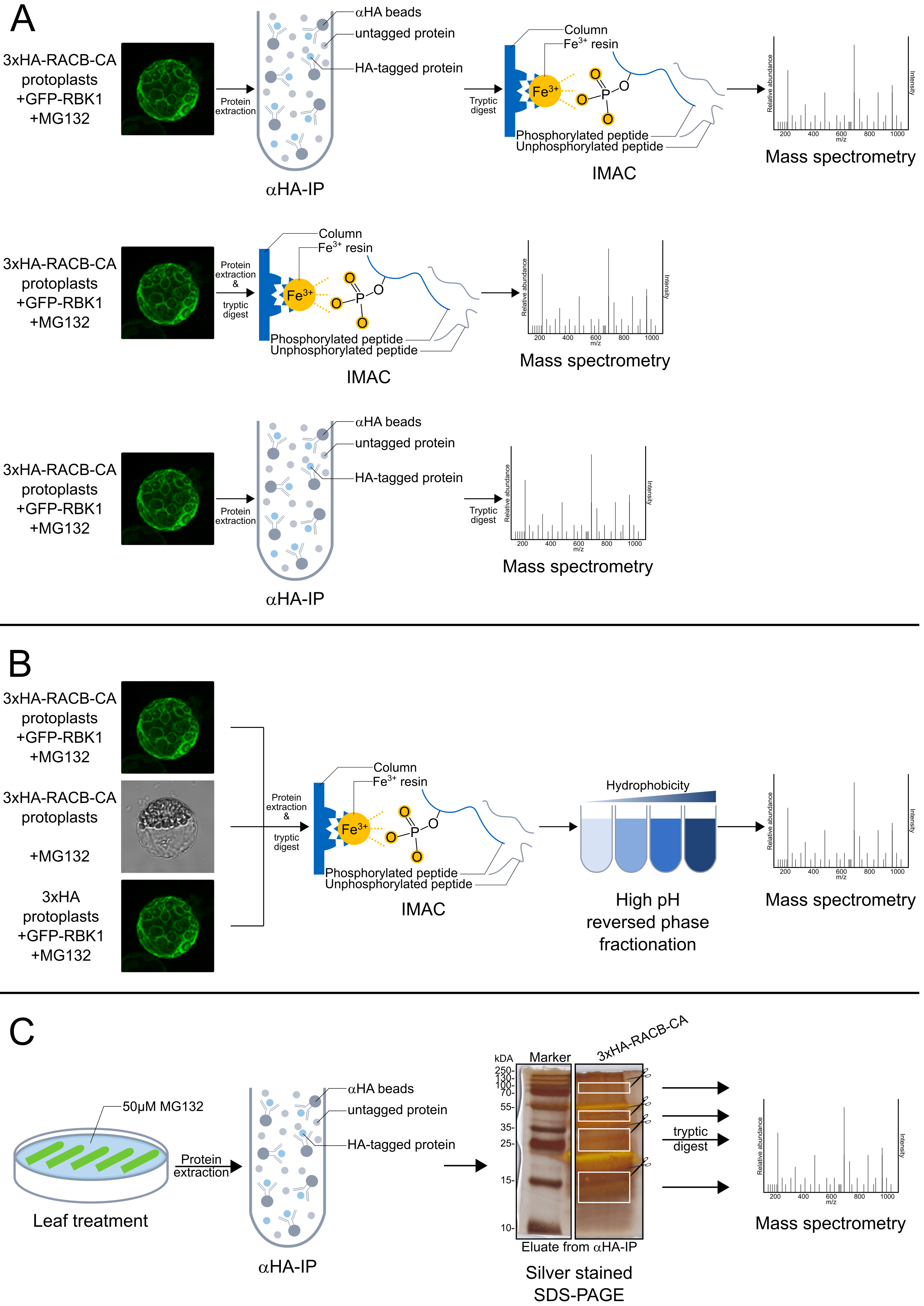

Supplement: S2 Fig — (A) Identification of phosphorylated peptides after CA-RACB and RBK1 overexpression. Protoplasts were isolated from stably-transgenic barley leaves overexpressing 3xHA-CA-RACB (pooled lines BG657 E3 and E4). These protoplasts were transiently super-transformed with GFP-RBK1 to stimulate phosphorylation events of proteins associated with the RACB/RBK1 pathway. After transformation, all protoplasts were treated with MG132 and proteins were extracted. HA-tagged proteins were first enriched using immunoprecipitation, followed by tryptic digest and phosphopeptide enrichment via IMAC (top panel). In two cases, immunoprecipitation or IMAC were skipped (center and lower panel, respectively). Phosphorylated peptides were identified using mass spectrometry. (B) Second experiment to identify phosphorylated peptides after CA-RACB and/or RBK1 overexpression. Protoplasts were isolated from stably transgenic barley leaves overexpressing either 3xHA (lines BG659 E2 and E3) or 3xHA-CA-RACB (lines BG657 E3 and E4). These protoplasts were super-transformed with GFP-RBK1. After transformation, all protoplasts (including an untransformed 3xHA-CA-RACB control) were treated with MG132. Proteins were extracted and digested with trypsin, followed by enrichment of phosphopeptides using IMAC. These samples were decomplexed by fractionating the phosphorylated proteins according to their hydrophobicity. Phosphorylated peptides were identified by mass spectrometry. (C) Analysis of CA-RACB ubiquitination by a targeted approach. Transgenic barley leaves overexpressing 3xHA-CA-RACB (from pooled lines BG657 E3 and E4) were floated on a solution containing MG132. HA-containing proteins were enriched by immunoprecipitation and loaded on a SDS-PAGE. Following silver-staining, RACB-containing bands and those corresponding to the higher molecular weight derivatives shown in Fig 4A were excised from the gel (white boxes). After solubilization and tryptic digest, modification of RACB-peptides was investiga [file pone.0258924.s002.tif]

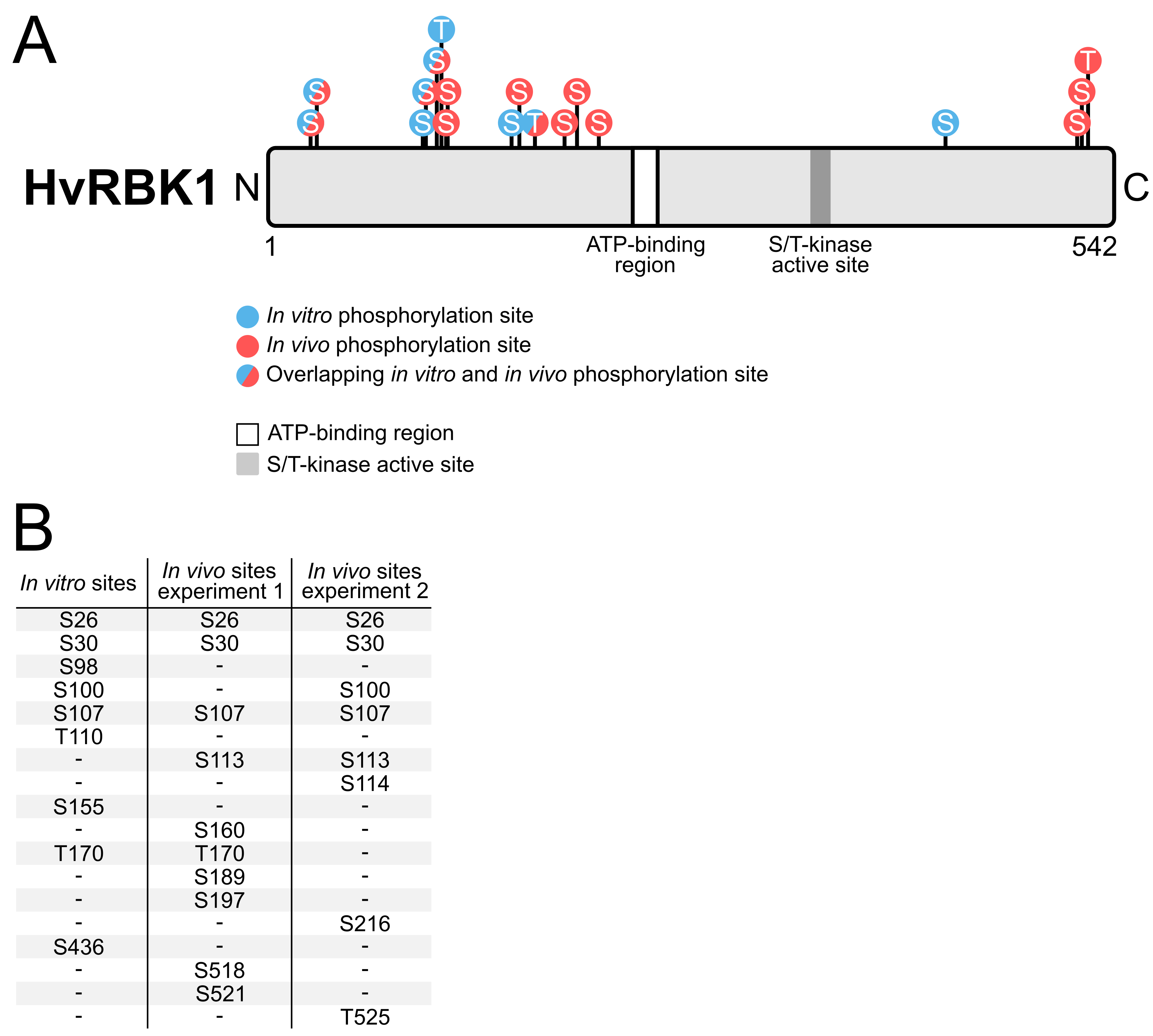

Supplement: S3 Fig — (A) Schematic illustration of the barley RBK1 sequence with marked in vitro (blue) and in vivo (red) phosphosites. Residues phosphorylated under both conditions are shown with both colors. In vitro phosphorylated amino acids were identified in an in vitro kinase assay, in which His-tagged RBK1 and His-tagged CA-RACB were co-incubated. In vivo phosphosites were found in transgenic HA-CA-RACB overexpressing mesophyll protoplasts in the presence of transiently co-overexpressed GFP-tagged RBK1 and the proteasome-inhibitor MG132. Phosphopeptides were enriched from total protein extracts of protoplast lysates using IMAC (experiment 1) and additionally decomplexed by high pH reversed-phase fractionation (experiment 2). All in vitro and in vivo phosphosites were identified by mass spectrometry. (B) List of RBK1 phosphosites from all in vitro and in vivo experiments. (TIF) [file pone.0258924.s003.tif]

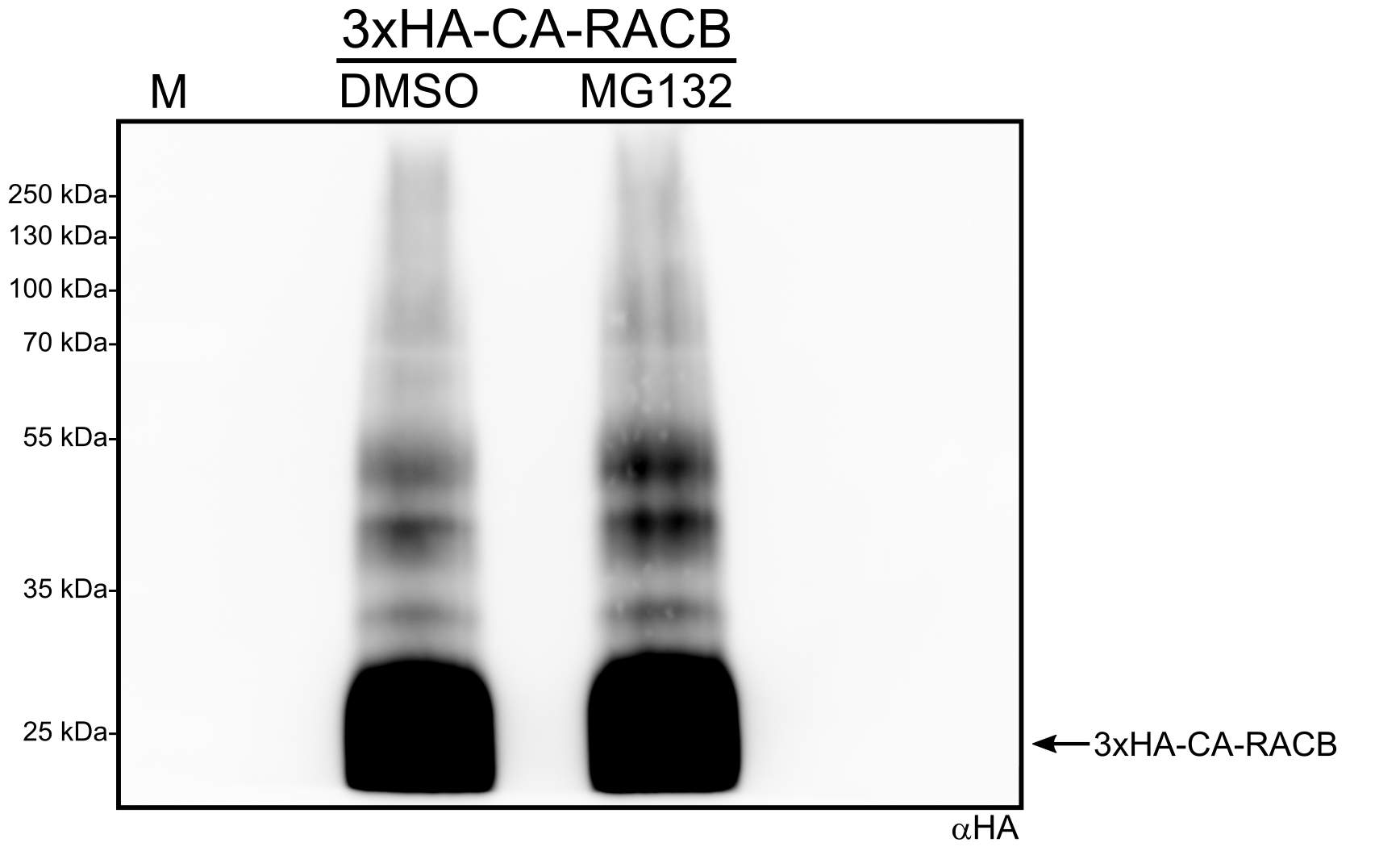

Supplement: S4 Fig — Whole first leaves of barley stably overexpressing 3xHA-CA-RACB were either floated on a solution containing MG132 or DMSO. After protein extraction, αHA-immunoprecipitation and αHA Western blotting, higher molecular weight laddering of 3xHA-CA-RACB could be detected independent of the treatment. Plant material originated from transgenic lines BG657 E3 and E4. (TIF) [file pone.0258924.s004.tif]

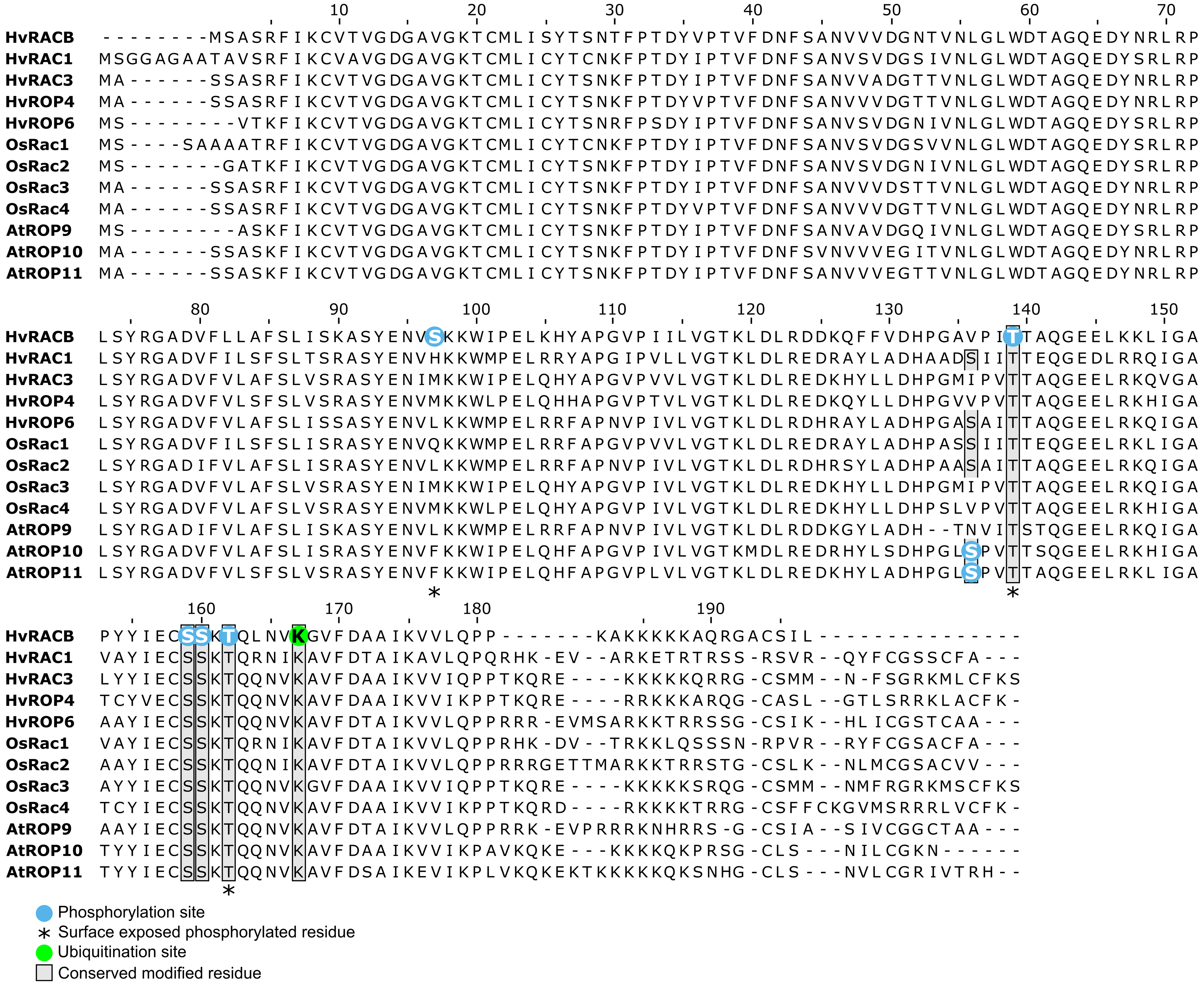

Supplement: S5 Fig — The CA-RACB ubiquitination site at K167 (green) is conserved in all type II ROPs from the three species (shaded box). In vitro phosphorylation sites of RACB (blue) are conserved among most type II ROPs (shaded box). Surface-exposed phosphosites are indicated by asterisks. Conserved amino acids were identified and aligned using MAFFT multiple-sequence alignment in Jalview [70, 71]. (TIF) [file pone.0258924.s005.tif]

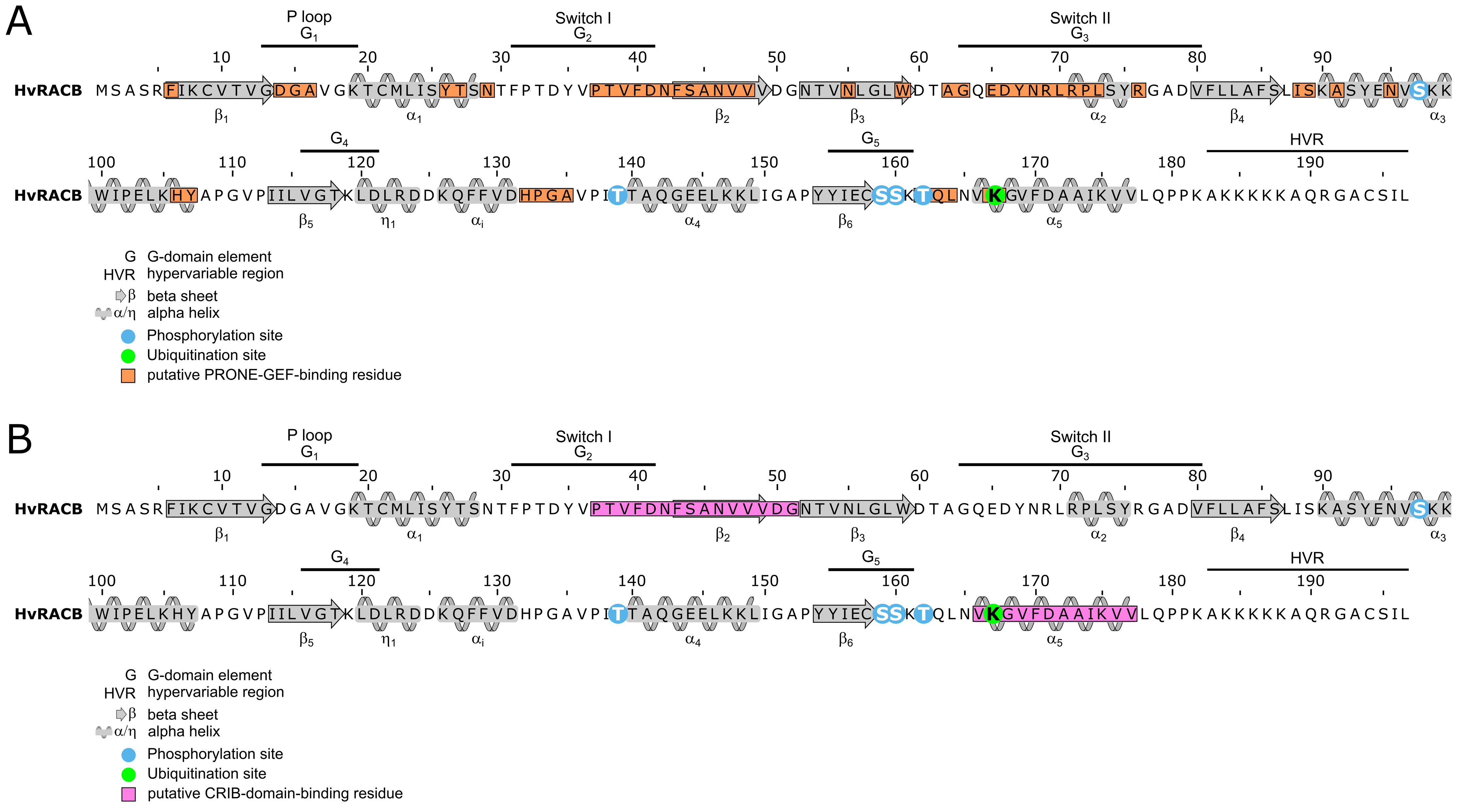

Supplement: S6 Fig — Predicted binding interfaces for PRONE-GEFs (A, orange boxes, [38]) and CRIB-domains (B, pink boxes, [56, 57]) are highlighted in the RACB amino acid sequence. RACB secondary structure motifs were obtained through homology-modelling using SWISS-MODEL with the crystal structure of AtROP5 as template (PDB: 3bwd.1, [67]). α-helices (grey boxes with winding helices) and β-sheets (grey arrows), as well as RACB in vitro phosphorylation (blue circle) and in vivo ubiquitination (green circle) sites are illustrated. Annotations for the G-domains (G1-5) and hypervariable region (HVR) were taken from [67], whereas regions for P loop and Switches I & II were adopted from [66]. Numbering for α-helices and β-sheets follow [38, 87]. (TIF) [file pone.0258924.s006.tif]
