# Supplementary material for: Posttranslational modification of the RHO of plants protein RACB by phosphorylation and cross-kingdom conserved ubiquitination
Source: PLoS One. 2022 Mar 25;17(3):e0258924. doi: 10.1371/journal.pone.0258924 (PMC8956194; doi:10.1371/journal.pone.0258924)
Supplement: S1 Raw images — (PDF) [file pone.0258924.s012.pdf]

Raw blots Fig1

His-RBK1 KD WT H_2O
RACB-CA-His - + - + - +

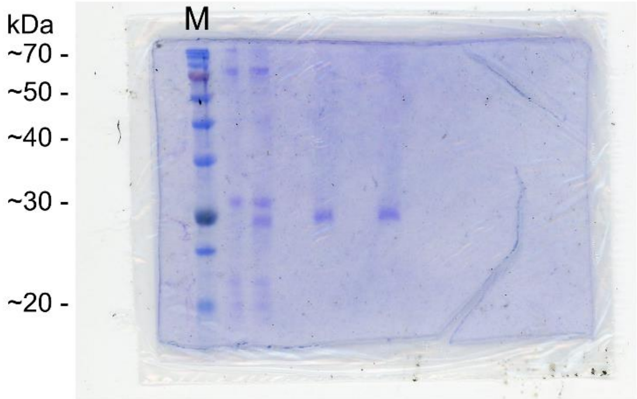

CBB protein stain

His-RBK1 KD WT H_2O
RACB-CA-His - + - + - +

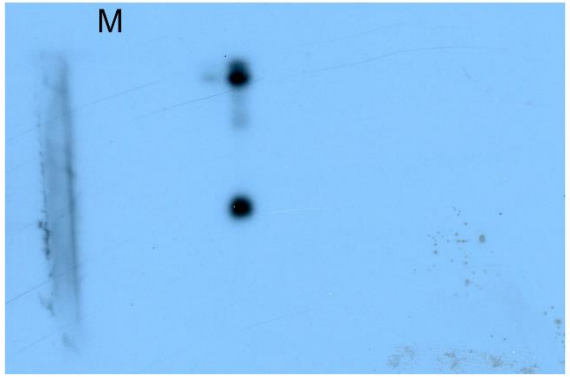

^{32}P autoradiogram

His-RBK1 KD WT

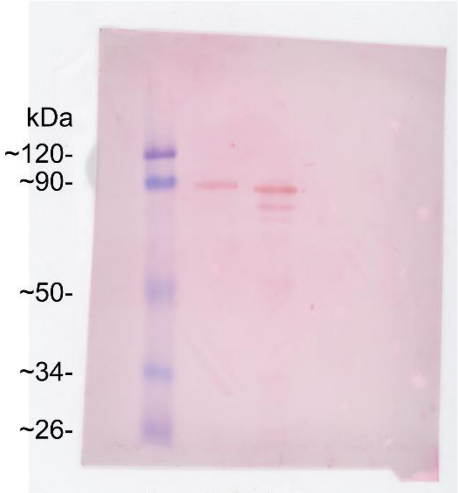

Ponceau S protein stain

Raw blots Fig3

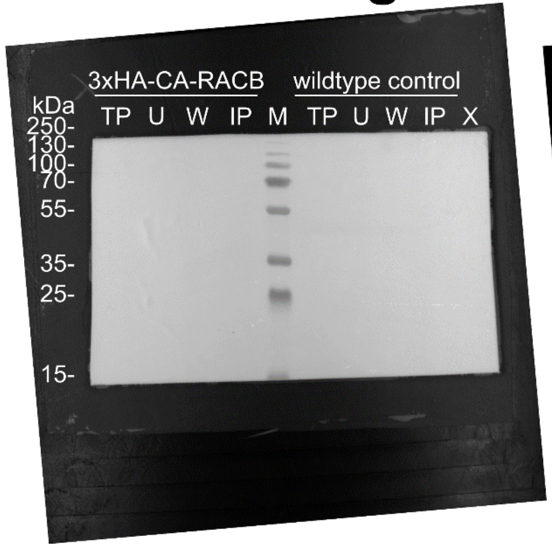

White light

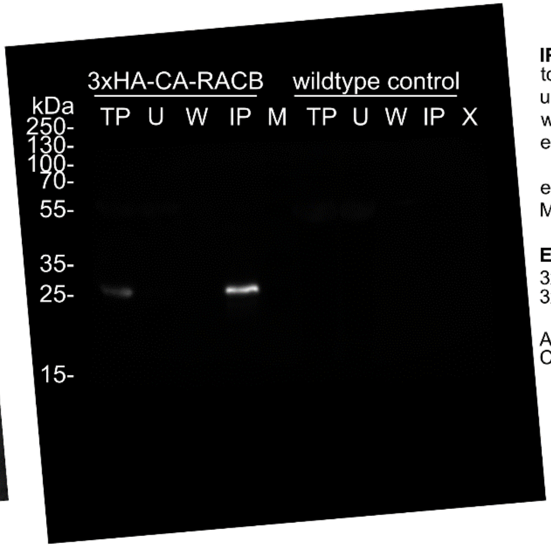

Chemiluminescence

IP fractions:
total protein (TP)
unbound flow-through (U)
wash (W)
eluate (IP)

empty lane (X)
Marker (M)

Estimated protein weights:
3xHA-CA-RACB: 25.1 kDa
3xHA-CA-RACB-ΔCSIL: 25 kDa

Antibody: anti-HA-HRP; Sigma
CCD-Camera: Fusion SL, Vilber-Lourmat

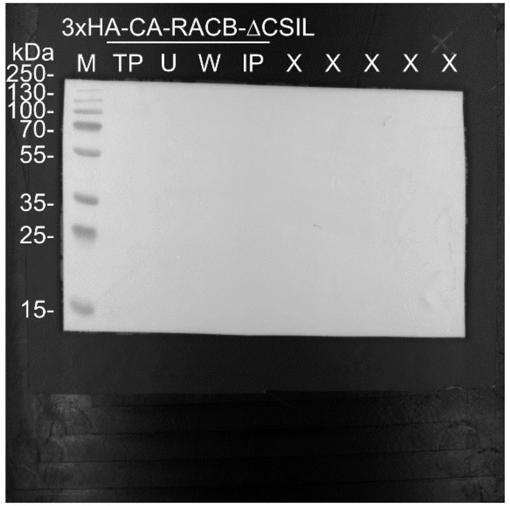

White light

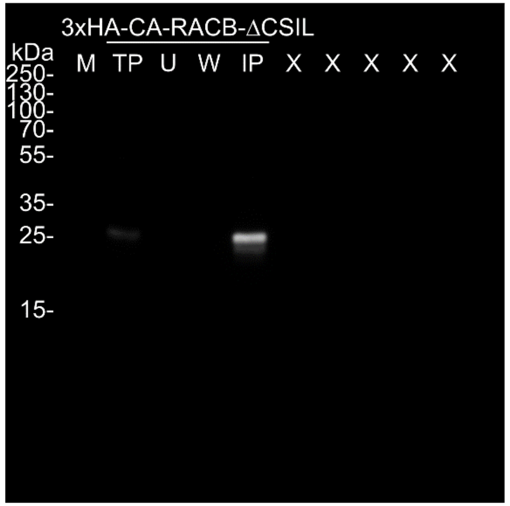

Chemiluminescence

Raw blots Fig5

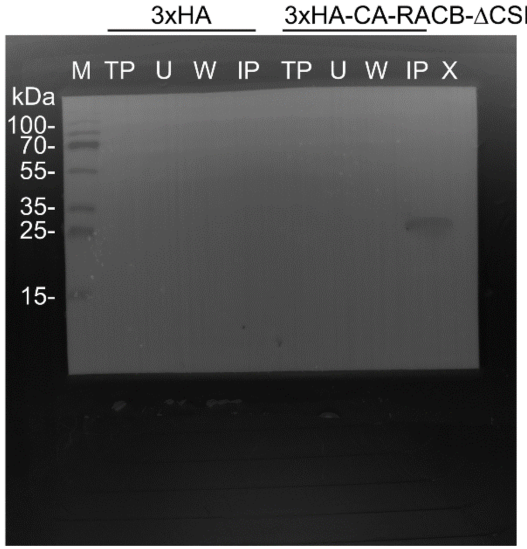

White light

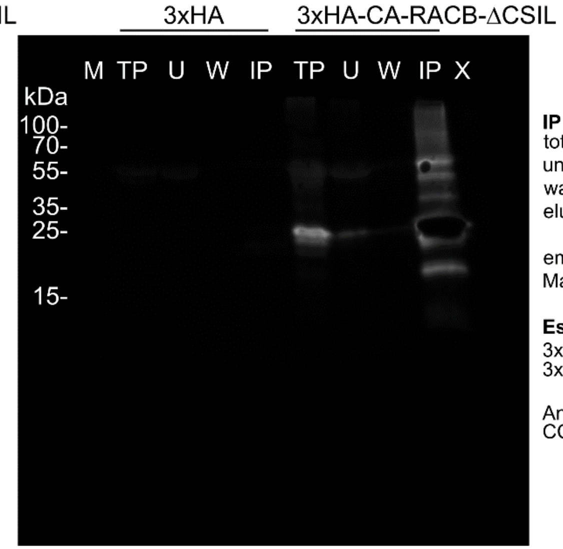

Chemiluminescence

IP fractions:
total protein (TP)
unbound flow-through (U)
wash (W)
eluate (IP)

empty lane (X)
Marker (M)

Estimated protein weights:
3xHA-CA-RACB: 25.1 kDa
3xHA-CA-RACB-ΔCSIL: 25 kDa

Antibody: anti-HA-HRP; Sigma
CCD-Camera: Fusion SL, Vilber-Lourmat

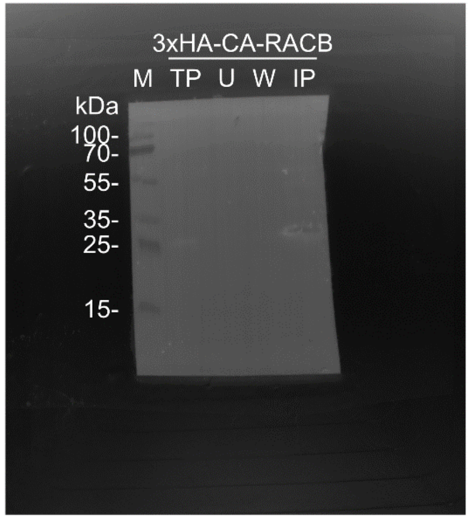

White light

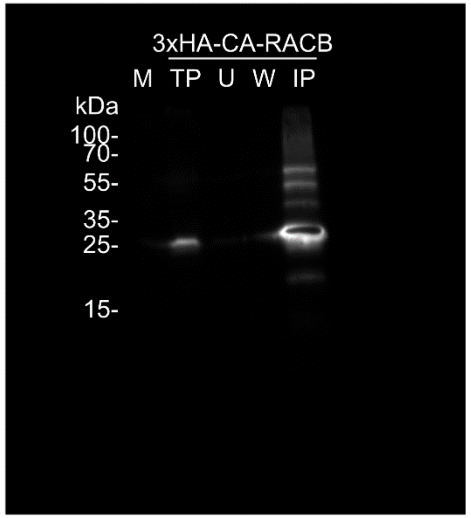

Chemiluminescence

Raw gel S2C Fig

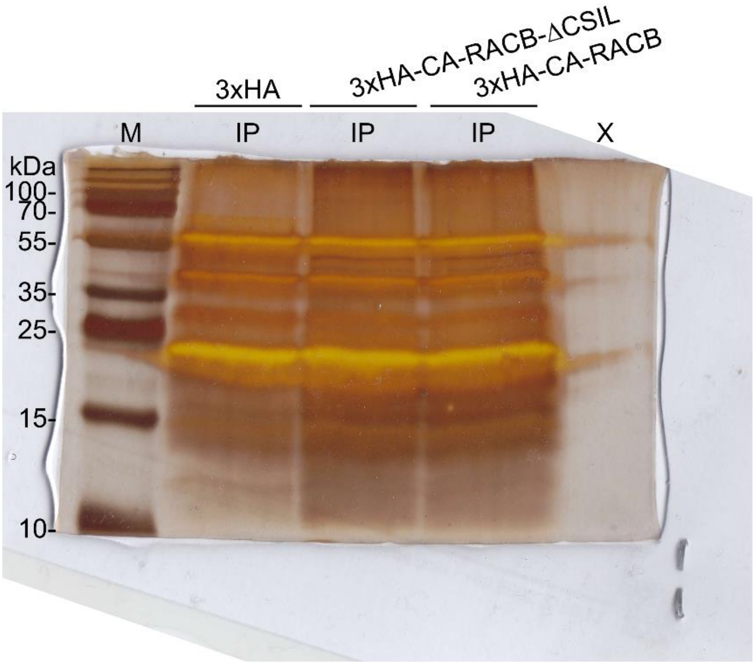

Silver-stained SDS-PAGE

IP fractions:
total protein (TP)
unbound flow-through (U)
wash (W)
eluate (IP)

empty lane (X)
Marker (M)

Estimated protein weights:
3xHA-CA-RACB: 25.1 kDa
3xHA-CA-RACB-ΔCSIL: 25 kDa

Scanned silver-stained SDS-PAGE

Raw blot S3 Fig

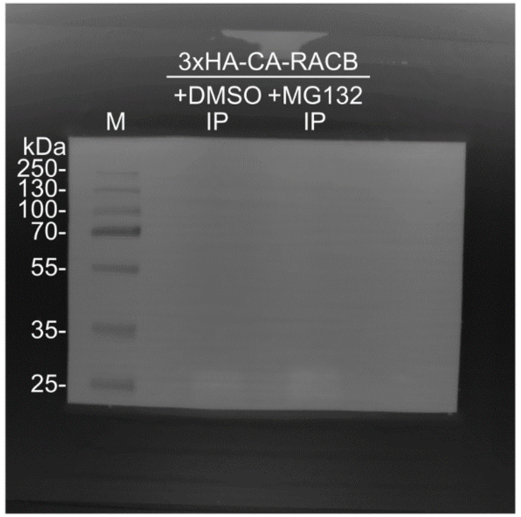

White light

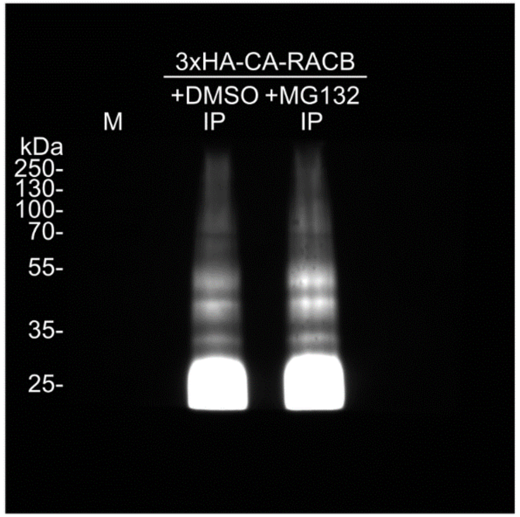

Chemiluminescence

IP fractions:
eluate (IP)

empty lane (X)
Marker (M)

Estimated protein weight:
3xHA-CA-RACB: 25.1 kDa

Antibody: anti-HA-HRP; Sigma
CCD-Camera: Fusion SL, Vilber-Lourmat
